# Autophagy controls the pathogenicity of *OPA1* mutations in dominant optic atrophy

**DOI:** 10.1111/jcmm.13149

**Published:** 2017-04-04

**Authors:** Mariame Selma Kane, Jennifer Alban, Valérie Desquiret‐Dumas, Naïg Gueguen, Layal Ishak, Marc Ferre, Patrizia Amati‐Bonneau, Vincent Procaccio, Dominique Bonneau, Guy Lenaers, Pascal Reynier, Arnaud Chevrollier

**Affiliations:** ^1^ PREMMi/Mitochondrial Medicine Research Centre Institut MITOVASC CNRS UMR 6015 INSERM U1083 Université d'Angers, CHU d'Angers Angers France; ^2^ Département de Biochimie et Génétique Centre Hospitalier Universitaire Angers France; ^3^ RGM4645 Université Blaise Pascal Aubière France

**Keywords:** optic atrophy, mitochondria, OPA1, mitophagy

## Abstract

Optic Atrophy 1 (OPA1) gene mutations cause diseases ranging from isolated dominant optic atrophy (DOA) to various multisystemic disorders. OPA1, a large GTPase belonging to the dynamin family, is involved in mitochondrial network dynamics. The majority of *OPA1* mutations encodes truncated forms of the protein and causes DOA through haploinsufficiency, whereas missense *OPA1* mutations are predicted to cause disease through deleterious dominant‐negative mechanisms. We used 3D imaging and biochemical analysis to explore autophagy and mitophagy in fibroblasts from seven patients harbouring *OPA1* mutations. We report new genotype–phenotype correlations between various types of *OPA1* mutation and mitophagy. Fibroblasts bearing dominant‐negative *OPA1* mutations showed increased autophagy and mitophagy in response to uncoupled oxidative phosphorylation. In contrast, OPA1 haploinsufficiency was correlated with a substantial reduction in mitochondrial turnover and autophagy, unless subjected to experimental mitochondrial injury. Our results indicate distinct alterations of mitochondrial physiology and turnover in cells with *OPA1* mutations, suggesting that the level and profile of OPA1 may regulate the rate of mitophagy.

## Introduction

Dominant optic atrophy (DOA) (Online Mendelian Inheritance in Man database #165500) is characterized by moderate to severe loss of visual acuity. The insidious onset of the disease in early childhood is caused by the selective loss of retinal ganglion cells (RGCs) and degeneration of the optic nerve. The majority of patients with genetically confirmed DOA (about 60–80%) harbour mutations in the *OPA1* gene coding for a mitochondrial GTPase. This dynamin GTPase mediates the fusion of mitochondrial inner membranes and is consequently a key moderator of the organization of the mitochondrial network. OPA1 is also involved in other functions including maintenance of the mitochondrial membrane potential 1, the integrity of the mitochondrial genome 2 and the control of apoptosis through its regulatory role in the organization of cristae and the subsequent trapping of cytochrome c 3. The *OPA1* gene consists of 30 coding exons covering a genomic region >60 kb. The gene is widely expressed, especially in the retina. The *OPA1* gene encodes a 960 amino acid polypeptide similar to specific GTP‐binding proteins of the dynamin protein family 4. A total of 377 *OPA1* gene variants have now been described 4, about 65% of which are considered pathogenic. Among the most frequent variants, 26% are missense, 16% frameshift, 14% non‐sense, 12% silent, and 7% deletion mutations 4. More than two‐thirds of the mutations are located in the dynamin and GTPase domains, the latter representing the most conserved part of the OPA1 protein. About 50% of the pathogenic variants result in the premature truncation of the open reading frame leading to OPA1 haploinsufficiency as a result of mRNA decay and complete loss of function of the mutated allele 5. The truncated transcripts, typically non‐sense and frameshift, often constitute less than the expected 50% of the total pool of *OPA1* transcripts. However, the extent of depletion of various mutant *OPA1* transcripts in ADOA patients is highly variable, ranging from no change at all to an apparent loss of about two‐thirds of the transcripts 5. Haploinsufficiency has been considered the main pathological mechanism involved in OPA1‐mediated disease. However, the recent emergence of severe multisystemic disorders, associated with missense mutations predominantly clustered in the GTPase domain, suggests that dominant‐negative or gain‐of‐function mutations may also play an important role in the pathological mechanism underlying DOA 6, 7, 8. The mutated proteins arising from these missense mutations, which cause heterozygous amino acid substitutions, are hypothesized to inhibit the wild‐type protein, thereby interfering with OPA1 functions 9. This hypothetical mechanism is further supported by a study of GTPase mutants of the merozoite surface protein 1 paralog (MSP1P), an intermembrane‐space dynamin‐related protein, in the wild‐type yeast Schizosaccharomyces pombe 10. The authors reported that a slight overexpression of mutated Msp1p in critical residues of the GTPase or GED domains in these wild‐type cells caused fragmentation of the mitochondrial network in about 60% of the cells.

The defining feature of DOA is optic atrophy, the severity of the visual loss being highly variable between and within families, ranging from the subclinical expression of the disease to legal blindness 11. The clinical spectrum has been further expanded to include chronic progressive external ophthalmoplegia and extraocular manifestations such as sensorineural deafness, ataxia, myopathy and peripheral neuropathy 6, 7. Other rare associations of *OPA1* mutations in adults have been reported with spastic paraplegia, the multiple sclerosis‐like syndrome, the adult Behr‐like syndrome, syndromic Parkinsonism, and dementia 12. In addition to these phenotypes associated with heterozygous *OPA1* mutations, early onset and severe syndromes due to biallelic *OPA1* mutations have been recently described 8, 13. Compound heterozygous *OPA1* mutations were found in patients with a Behr‐like syndrome (MIM#210000), associating spinocerebellar degeneration, pyramidal signs, peripheral neuropathy, gastrointestinal dysmotility and retarded development 8. This high phenotypic diversity, ranging from moderate and isolated DOA to severe neonatal syndromes, is related both to the mono‐ or bi‐allelic inheritance as well as to the type of *OPA1* mutation, the risk of the occurrence of DOA+ being significantly greater in the case of a missense mutation than in that of a truncating mutation 14.

The diversity of the pathophysiological mechanisms involved in *OPA1*‐related disorders is linked to the crucial role played by OPA1 in the maintenance of mitochondrial structure, genome and function 15, 16, 17. Through its pro‐fusion activity, OPA1 dysfunction has various deleterious consequences that have been reported in several models and patients, such as increased susceptibility to apoptosis 1, 18, defective bioenergetics 19, altered calcium homoeostasis 20, compromised synaptic maturation, and dendritogenesis 21. How specific *OPA1* mutants affect these functions has been widely investigated but remains without consensus, as the question of whether *OPA1* mutations lead to the selective degeneration of RGCs and eventually to that of other neurons remains unanswered. The mechanism currently proposed is that *OPA1* mutations sensitize cells to mitochondrial apoptosis 3, 22, but some studies have found no evidence to support this hypothesis either in human cells harbouring *OPA1* mutations or in other ADOA‐linked animal models 23. Indeed, in Caenorhabditis elegans, mutations of the *OPA1* gene orthologue, eat‐3, showed no evidence of increased cell death 24. Moreover *OPA1*‐/‐ MEFs (mouse embryonic fibroblasts) were observed to be more resistant to pro‐apoptotic treatment with staurosporine when compared to their wild‐type counterparts 17. Nonetheless, it cannot be excluded that reduced OPA1 functions may sensitize cells to other forms of cell death pathways such as autophagic cell death 25. As autophagy constitutes a cellular survival pathway, which recycles cellular components and ensures the appropriate degradation of organelles, it is conceivable that constitutive autophagy leads to cell death. The selective degradation of damaged mitochondria by autophagy is known as mitophagy. The presence of autophagic vesicles in RGCs and in various other tissues of the DOA mouse model supports the hypothesis of RGC loss by programmed autophagic cell death 26, 27. The alteration of mitophagy, a component of mitochondrial quality control, has been closely associated with neurodegeneration 28. Several lines of evidence suggest a tight bidirectional regulation between the autophagic machinery and mitochondria through stimuli such as modified energy levels and mitochondrial dynamics 29. Indeed, studies of the mitophagic process have shown that the targeting of damaged, depolarized mitochondria by lysosomes, requires the fission of the mitochondrial network 30. OPA1, which plays a role in the fusion of the mitochondrial network, may be involved in the modulation of mitophagy. The considerable variability in the expression of DOA revealed by studies of *OPA1* mutations has been attributed to the type or position of the mutations. In this study, we investigate a cohort of patients harbouring missense, non‐sense or bi‐allelic *OPA1* mutations to examine the role played by mitophagy and autophagy in the physiopathology of DOA.

## Materials and methods

### Patients

Written, informed consent was obtained from all patients participating in this study. Approval for this work was granted by the Ethics Committee of the University Hospital of Angers (*Comité de Protection des Personnes CPP Ouest II* – Angers, France; Identification number CPP CB 2014/02; Declaration number DC‐2011‐1467 and Authorization number AC‐2012‐1507). Five healthy age‐matched individuals, with no signs of optic atrophy, served as controls. The characteristics of the patients are shown in Table 1; c.1146A>G/p.I382M on transcript NM_015560 is also referenced as c.1311A>G/p.I437M, NM_130837.1.

**Table 1:** Clinical and molecular description of patients with *OPA1* mutations

Patients (Gender, Age)	Age of onsetOpticAtrophy	Ataxia	Neuropathy	Digestive symptoms	Others	Mutations in OPA1	Exon/Domain	PROVEAN	Polyphen2	Sift	MutationTaster	FATHMM	Ref.
Patient 1 (M, 42 years)	3 years	+	−	−	−	p.Ser545Arg	Exon 17 (Dynamin) c.1635 C>G	−4.585 *Deleterious*	0.991 probably damaging	0 DAMAGING	110 disease causing	−3.59 DAMAGING	7
Patient 2 (M, 49 years)	5 years	+	−	−	−	p.Ser545Arg	Exon 17 (Dynamin) c.1635 C>G	−4.585 *Deleterious*	0.991 probably damaging	0 DAMAGING	110 disease causing	−3.59 DAMAGING	7
Patient 3 (F, 30 years)	6 years	−	−	−	Deafness	p.Arg445His	Exon 14 (GTPase) c.1334 G>A	−4.701 *Deleterious*	0.966 probably damaging	0 DAMAGING	29 disease causing	−4.21 DAMAGING	6
Patient 4 (M, 14 years)	18 months	+	+ peripheral neuropathy	+	Cerebellar Atrophy	p.Ile382Met	Exon 12 (GTPase) c.1146 A>G	−2.820 *Deleterious*	0.999 probably damaging	0 DAMAGING	10 disease causing	−4.44 DAMAGING	8
						p.Arg824*	Exon 24 (Dynamin) c.2470 C>T	*Truncation*	Truncation	Truncation	6 disease causing	Truncation	
Patient 5 (F, 4 years)	14 months	+	+ peripheral neuropathy	+	−	p.Ile382Met	Exon 12 (GTPase) c.1146 A>G	−2.820 *Deleterious*	0.999 probably damaging	0 DAMAGING	10 disease causing	−4.44 DAMAGING	8
						p.Arg557*	Exon 17 (GTPase) c.1669 C>T	*Truncation*	Truncation	Truncation	6 disease causing	Truncation	
Patient 6 (M, 15 years)	36 months	+	+	−	Vermian Atrophy	p.Ile382Met	Exon 12 (GTPase) c.1146 A>G	−2.820 *Deleterious*	0.999 probably damaging	0 DAMAGING	10 disease causing	−4.44 DAMAGING	8
						p.Glu487Lys	Exon 15 (GTPase) c.1459 G>A	−2.488 *Neutral*	0.411 benign	0.03 DAMAGING	56 disease causing	−3.65 DAMAGING	
Patient 7 (M, 16 years)	12 months	+	+	−	Epilepsy	p.Ile382Met	Exon 12 (GTPase) c.1146 A>G	−2.820 *Deleterious*	0.999 probably damaging	0 DAMAGING	10 disease causing	−4.44 DAMAGING	−

M, male; F, female. * : Stop.

### Cell cultures and reagents

Primary fibroblast cultures were established using standard procedures in 2/3 Dulbecco's modified Eagle's medium with 4.5 g/ml glucose and 1/3 AmnioMax (Thermo Fisher Scientific, Waltham, MA, USA), supplemented with 10% foetal bovine serum (FBS), 5 mM uridine (Sigma, Saint‐Louis, MO, USA) and 10 mM pyruvate (Sigma). The cultures were incubated at 37°C with 5% CO_2_. Cells were used at approximately 80% confluency and at fewer than 20 passages. Cells were treated with dimethylsulfoxide (Sigma) vehicles, 60 μM chloroquine diphosphate salt (Sigma), and 10 μM carbonyl cyanide 3‐chlorophenylhydrazone (Sigma) (durations of treatment are indicated in the figure legends).

### Mitochondrial morphology

Fibroblasts were seeded either on Lab‐tek glass coverslips (Thermo Fisher Scientific) at least one day before imaging, or on micropatterned coverslips (Cytoo^®^) (Cytoo SA, Grenoble, France) at least 4 hrs before image acquisitions. Mitochondrial morphology was assessed by staining cells with 10 nM Mitotracker Red (Life Technologies, Carlsbad, CA, USA) for 15 min. at 37°C. Wide‐field cellular fluorescence images were acquired with an inverted Leica microscope (Leica Microsystems, Wetzlan, Germany) equipped with a Roper CoolSnap HQ2 camera (Roper Scientific, Munich, Germany). Images were collected using a × 100/1.5 oil objective. Data were acquired and analysed using Metamorph software^®^ 7.7 (Universal Imaging Corporation, Downingtown, PA, USA). An average of 21 image planes was acquired along the *z*‐axis with an increment of 0.2 μm between each step. Measurements were performed at room temperature (20°C). During acquisition and image data analysis, only well‐spread triangular cells were selected to minimize biases arising from the variation of cell size and shape 19. An average of 100 cell acquisitions from micropatterned slides was used. The images were deconvolved with Huygens Essential Software (Scientific Volume Imaging, Hilversum, Netherlands). All the selected images were iteratively deconvolved with a maximum iteration scored 50 and a quality threshold of 0.05. Bitplane's Imaris 7.1.1^®^ software (Bitplane, Zurich, Switzerland) was used for 3D processing and morphometric analysis.

### Fluorescence microscopy and quantitative analysis of mitophagy

For mitophagy experiments, cells were loaded with Lyso‐ID (Enzo Life Technologies, Farmingdale, NY, USA) to mark lysosomal structures (green) and MitoTracker Red FM (Life Technologies) to visualize mitochondria (red) for 30 min. The degree of colocalization of mitochondria with lysosomes was measured *via* live cell imaging microscopy at 37°C in an atmosphere of 5% CO_2_, using an average of 100 cell acquisitions from micropatterned slides. Bitplane's Imaris 7.1.1^®^ software was used for analysis. Spot detection software and surface tools were used for the 3D detection of lysosome and autophagosome structures (see Movie S1). Contacts between these structures and mitochondria, considered as markers of cell degradation, were determined using the colocalization tool provided with Bitlane's Imaris 7.1.1^®^ software. Complete mitochondrial and lysosomal staining were noted as regions of interest and subjected to colocalization analysis with the threshold setting in channel 1 (red) and channel 2 (green). Finally, object‐based colocalization analysis by fluorescence intensity profiles was used to measure the colocalization of LysoID and MitoTracker Red fluorescence.

### Immunoblot analysis

For immunoblotting, cells were lysed in a buffer containing 10 mM Tris pH 7.4, 150 mM NaCl, 1% Triton X‐100, 10% glycerol, 10 mM EDTA, and the protease inhibitor cocktail (Roche Applied Science, Mannheim, Germany). After 20 min. rotation at 4°C, the lysates were centrifuged at 10,000 × *g* at 4°C for 10 min. Protein extracts (30–60 μg) were separated on 4–20% Tris‐Glycine Gel (Life Technologies) and electron‐transferred to polyvinylidene difluoride or nitrocellulose membranes according to standard procedures. After blocking free binding sites with 5% non‐fat dry milk reconstituted in Tris‐buffered saline with 0.2% Tween‐20, the membranes were probed with various antibodies: mouse anti‐LC3B (1/2000, Enzo, #ALX‐803‐081‐C100) (Enzo‐life Sciences, Lyon, France), rabbit anti‐P62 (1/1000, Enzo, #BMLPW9860‐0100) mouse anti‐ P84 (1/1000, Abcam, #ab487), mouse anti‐VDAC (1/2000, Abcam, #ab34726), mouse anti‐Human Oxphos (1/1000, Abcam, #ab110411) and mouse anti‐tubulin (1/2000, Sigma, #T9026). Anti‐mouse and anti‐rabbit peroxidase‐linked secondary antibodies (GE Healthcare, Life Sciences) were used at 1/10,000 and 1/20,000 respectively, and signals were detected using a chemiluminescence system (Super signal^®^ West Femto, Thermo Scientific, #34095) (Thermo Fisher Scientific). The images acquired were analysed quantitatively using Image Studio 2.1 software (Li‐Cor, Lincoln, NE, USA).

### Electron microscopy

The samples used for transmission electron microscopy were processed using standard protocols. Fibroblasts from DOA patients and controls were collected and fixed with 2.5% glutaraldehyde in 0.1 M sodium cacodylate buffer (pH 7.4) at 37°C. Following fixation, samples were placed in 2% osmium tetroxide in 0.1 M sodium cacodylate buffer (pH 7.4), dehydrated in graded series of ethyl alcohol and embedded in Durcupan resin (Sigma‐Aldrich, Saint‐Louis, MO, USA). Ultrathin sections were cut with an ultramicrotome and placed on grids. Following counterstaining, images were acquired with an AMT XR‐60 CCD camera system (AMT, Woburn, MA, USA). The number of autophagic vesicles per cell area was estimated using square grid openings, which were selected according to the rules of uniform random sampling 31.

### Measurement of oxygen consumption

Respiration rates were assessed on intact cells (1.1*10^6^ cells) suspended in DMEM‐F12, as described elsewhere 19. Cellular oxygen consumption was measured at 37°C on a high‐resolution oxygraph (Oroboros, Innsbuck, Austria). Baseline respiration was measured in the absence of exogenous substrates, whereas non‐phosphorylating respiration was achieved by addition of oligomycin at a concentration of 2 μg/ml. In order to see how the patients’ cells reacted to an increased demand of ATP, the uncoupler carbonyl‐4‐(trifluoromethoxy)‐phenylhydrazone (FCCP), was added to dissipate the electrochemical gradient that drives ATP synthesis. This maximal respiration was accomplished by a stepwise manual titration of 4 mM FCCP. Finally, mitochondrial‐specific respiration was controlled by the inhibition of cytochrome bc1 (complex III) with 2.5 μM antimycin.

### Quantification of mitochondrial DNA

Mitochondrial DNA (mtDNA) was quantified both through quantitative PCR and the live microscopy approach as mtDNA in cells is organized in nucleoids containing DNA and various proteins. To assess mtDNA copy number through quantitative PCR, total DNA was extracted with the Roche Kit according to manufacturer's guidelines. The quantification of mtDNA corresponds to the qPCR ratio obtained with the use of two primers specific to mitochondrial DNA, and two primers specific to nuclear genes, using iQ SYBR Green Supermix and the Chromo 4 Real‐Time PCR Detection System (Bio‐Rad). The primers’ information were as follows: MitoDNA_RNAL: Forward 5′‐CGCATAAAACTTAAAACTTTACAG‐3′; Reverse 5′‐CTTTGCGTAGTTGTATATAGC‐3′; MitoDNA_ND4: Forward 5′‐CAGCCACATAGCCCTCGTAG‐3′; Reverse 5′‐GCGAGGTTAGCGAGGCTTGC‐3′; Nuclβ‐glob Forward TTGTCTTTCAGCAAGGACTG‐3′; Reverse ATCTTGGGCTGTGACAAAGT‐3′; NuclAIB: Forward 5′‐GGAGTTTCCTGGACAAATGA; Reverse 5′‐AGGACTGGCGTTTATGTCTT‐3′. The thermal cycler conditions consisted of 95°C for 3 min., followed by cycles of 95°C for 15 sec. and 59°C for 40 sec. The analyses were performed in triplicates.

To quantify mtDNA content through live microscopy, nucleoids were stained by diluting stock PicoGreen^®^ solution (Molecular Probes, Eugene, OR, USA) at 2 μl/ml directly into the cell culture medium for 15 min. Co‐staining was performed with a mitochondrion‐selective dye, tetramethylrhodamine methyl ester perchlorate^®^ (TMRM) at 50 nM for 15 min. Image treatment was performed using Imaris spot detection software (Bitplane) to visualize and quantify mtDNA/nucleoid labelling. An average of 50 cell acquisitions from micropatterned slides was used.

### Statistical analysis

At least three biological replicates of each fibroblast cell line were analysed. The values of mutant cell lines compared to control cells that were beyond ±2 SD (standard deviation) to the mean were considered to be significantly different.

## Results

### *OPA1* mutations and the protein profile of OPA1

**Figure 1:**
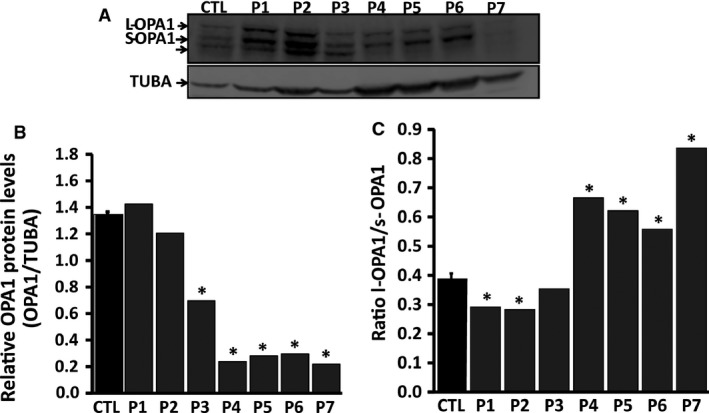
Immunoblot analysis of OPA1 expression in *OPA1* mutant fibroblasts and controls. The protein extracts were obtained from primary cell cultures. Cells were directly quenched with the same buffer as used for cell lysis (see Materials and methods) at 4°C to avoid cleavage of long‐OPA1 into short‐OPA1 isoforms. (**A**) Immunoblot bands of OPA1 protein. (**B**) Densito‐metrical analysis of the protein bands shown in A); the figure shows ratios of densities of the protein band to densities of the corresponding tubulin alpha band (TUBA) bands. (**C**) The figure shows ratios of the long (l‐OPA1) to the short (s‐OPA1) OPA1 isoforms. Data obtained from six independent experiments were analysed. P1‐P7: patients; CTL: controls.

*OPA1* mutations are grouped into two major categories depending on whether they are predicted to cause disease because of haploinsufficiency (deletions, splice site and non‐sense mutations) or through a possible dominant‐negative mechanism (missense mutations) 14. However, some missense mutations have also been reported to lead to haploinsufficiency 12. We further investigated whether the *OPA1* mutations result in lower OPA1 protein levels, or whether some compensatory mechanisms counteract the genetic defect, especially in a pooled sample of bi‐allelic cases carrying the p.I382M mutation, as few studies have focused on this variant. Western blot analysis with whole‐cell lysates of primary fibroblasts harbouring compound heterozygous *OPA1* mutations with the co‐occurrence of the missense I382M mutation and a non‐sense *OPA1* mutation revealed a decreased amount of OPA1 protein normalized for tubulin in patients 4–6, suggesting that haploinsufficiency contributes to the pathogenesis (Fig. 1A,B). Surprisingly, fibroblasts from one of the patients harbouring only the I382M mutation displayed a pattern similar to that observed in fibroblasts from patients with the bi‐allelic *OPA1* mutation. Indeed, fibroblasts from Patient 7 manifested a >80% loss of the OPA1 protein compared to a mean loss of 65% in compound heterozygous fibroblasts (Fig. 1B). These findings resemble those previously reported in fibroblasts from a patient carrying the same I382M mutation 32. Moreover, the deleterious score of the I382M mutation reinforces the pathogenic character of this variant (Table 1). However, fibroblasts from patients harbouring the p.S545R mutation (patients 1 and 2) and the p.R445H mutation (Patient 3) showed no significant alteration in OPA1 protein levels compared to control fibroblasts (Fig. 1B).

*OPA1* gene expression produces many isoforms through differential splicing with mRNA forming a long isoform and several short isoforms 33. Western blot analysis showed the loss of one short isoform with a subsequent increase in the ratio of long‐OPA1/short‐OPA1 (patients 4–7) (Fig. 1C). The protein profiles of OPA1 in the p.S545R and p.R445H mutations were similar those of the controls, despite the lower ratio of long‐OPA1 to short‐OPA1 (Fig. 1C). Taken together, these findings led us to discriminate between two groups of *OPA1* mutations, that is dominant‐negative *OPA1* mutations, which have no effect on OPA1 protein levels, and haploinsufficient *OPA1* mutations associated with a reduction in the amount of *OPA1* protein.

### Morphological and biochemical analysis of fibroblast cell lines

**Figure 2:**
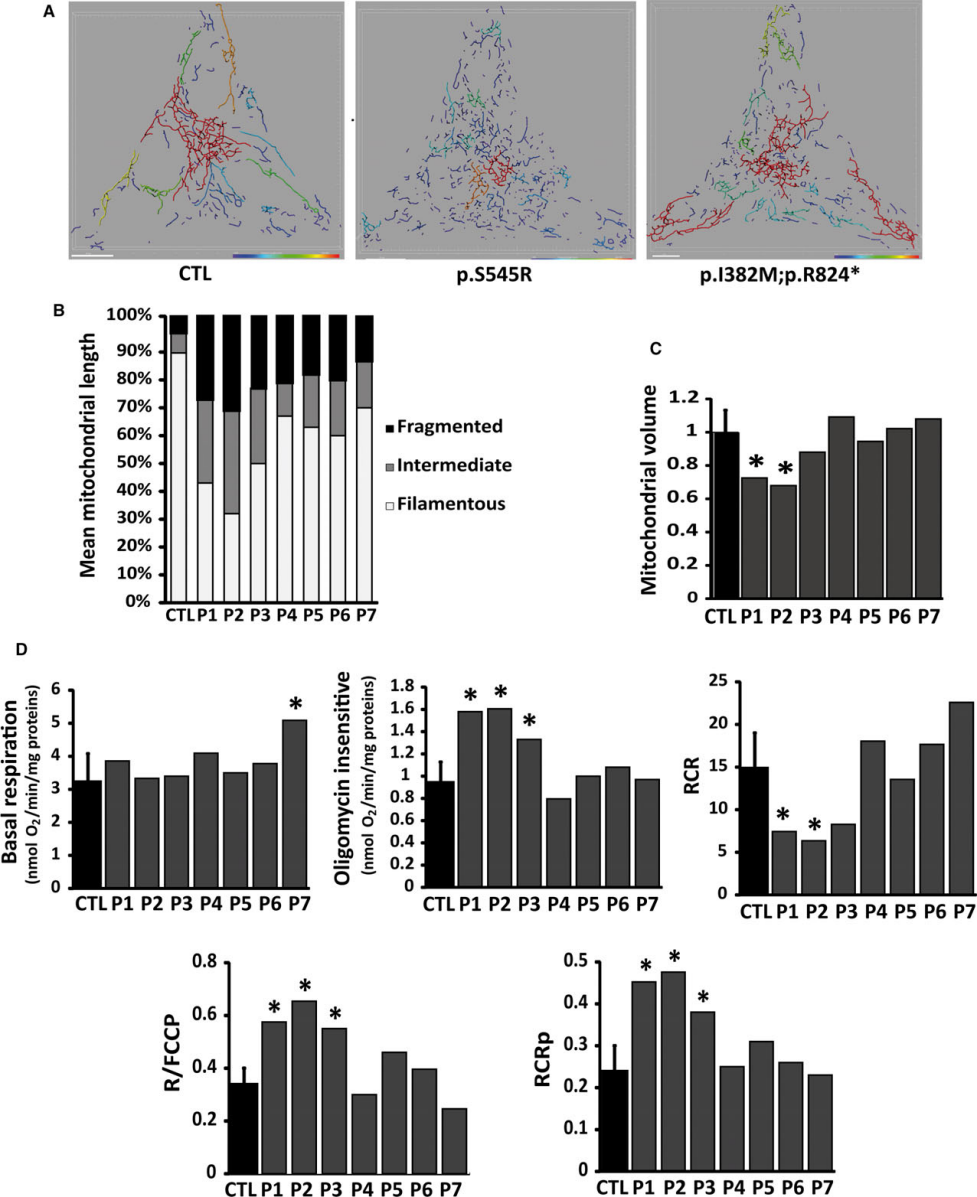
Morphology of mitochondrial network and bioenergetics in control and *OPA1* mutated fibroblasts. Cell volumes and shapes were standardized using micropatterned coverslips. Mitochondrial lengths were assessed and colour‐coded (*e.g*. the red colour represents mitochondria >20 μm). (**A**) Fibroblasts were incubated in 2/3 Dulbecco's modified Eagle medium and 1/3 Amniomax for 48 hrs, and then loaded with MitoTracker Red as described in Materials and methods. Representatives of 5–10 similar images are shown for each cell line. Scale bar = 10 μm. (**B**) Bar graphs show the distribution of the mitochondrial population in three different categories on the basis of mitochondrial morphology: ‘filamentous’, mitochondria >20 μm; ‘intermediate’, mitochondria 5–20 μm; and ‘fragmented’, mitochondria <5 μm. (**C**) Bar graphs show mitochondrial volume quantified as described in Materials and methods. Data were obtained from three independent experiments. (**D**) Mitochondrial oxidative phosphorylation was analysed on control (CTL) and *OPA1* mutated fibroblasts. Oxygen consumption was measured under basal conditions (Basal respiration graph), in the presence of oligomycin (4 μg/ml) (oligomycin‐insensitive respiration graph), and in the presence of FCCP (4 mM). The respiratory control ratio (RCR graph) was calculated as the ratio of the FCCP‐uncoupled to the oligomycin‐insensitive respiration. Respiration coupled to ATP production (RCRp graph) was calculated as the ratio of the difference between the rates of basal and oligomycin‐insensitive respiration to the uncoupled FCCP respiration. Respiratory reserve capacity was calculated as the ratio of basal respiration to the FCCP‐uncoupled respiration (R/FCCP graph). Data obtained from six independent experiments were analysed. P1‐P7: patients; CTL: controls.

Mitochondrial network morphology was investigated in fibroblasts bearing *OPA1* mutations and control fibroblasts maintained in a glucose medium supplemented with uridine and pyruvate. The fibroblasts were classified in three categories on the basis of mitochondrial morphology, that is cells with filamentous and interconnected mitochondria, cells with coexisting long and short mitochondria, and cells with completely fragmented mitochondria (Fig. 2A). Whereas control fibroblasts maintained a filamentous and interconnected mitochondrial network, *OPA1* mutant fibroblasts displayed various stages of mitochondrial fragmentation (Fig. 2B and Fig. S1). Indeed, in contrast to fibroblasts bearing the I382M mutation, which displayed mild mitochondrial network fragmentation, fibroblasts with the p.R445H and p.S545R mutations were marked by the disappearance of filamentous mitochondria in favour of fragmented and punctuated mitochondria (patients 1–3) (Fig. 2B and Fig. S1). Quantification of the ratio of mitochondrial to cellular volume showed a 20% decrease of mitochondrial content in fibroblasts with the dominant‐negative p.R445H and p.S545R mutations (patients 1–3), compared to control fibroblasts, whereas no significant loss of mitochondrial volume was observed in the haploinsufficient *OPA1* mutated fibroblasts (patients 4–7) (Fig. 2C). The mitochondrial content was further assessed by measurement of mitochondrial DNA copy number by quantitative real‐time PCR and live‐staining of nucleoids (Fig. S2). Quantification by qPCR approach do not show mitochondrial DNA content variation but fluorescent imaging by co‐staining with Picogreen and TMRM showed a 25% decrease number of nucleoid in P1‐3, compared to control fibroblasts (Fig. S2C). These approaches were combined with immunofluorescence techniques quantifying porin; the mitochondrial voltage‐dependent anion channel, and oxidative phosphorylation subunits (Fig. S2D), which also indicated a reduction in mitochondrial content in some *OPA1‐*mutated fibroblasts.

As *OPA1* down‐regulation has been reported to impinge on mitochondrial respiration, we evaluated the mitochondrial respiratory function on intact *OPA1‐*mutated fibroblasts. In *OPA1‐* mutated fibroblasts as well as in controls, oligomycin inhibited ATP‐linked respiration, while addition of FCCP, an uncoupling agent, led to maximal respiration. Dominant‐negative *OPA1‐*mutated fibroblasts (patients 1–3) showed a lower respiratory control ratio (RCR) with a slight increase of oligomycin‐insensitive respiration compared to haploinsufficient *OPA1‐*mutated fibroblasts (patients 4–7) and controls, indicating an increased proton leak (Fig. 2D). The respiration coupled to ATP production (RCRp) and the ratio of basal respiration to the FCCP‐uncoupled respiration (R/FCCP) were both greater in fibroblasts from patients 1–3 than in controls. This indicates that dominant‐negative *OPA1‐*mutated fibroblasts use a greater portion of their respiratory reserve capacity than other *OPA1‐*mutated fibroblasts and controls to sustain routine respiration (Fig. 2D). In addition, we found mild uncoupling in fibroblasts with the p.R445H and the p.S545R mutations whereas haploinsufficient *OPA1‐*mutated fibroblasts were similar to control fibroblasts, showing no alteration of mitochondrial respiration. These findings show that dominant‐negative *OPA1‐*mutated fibroblasts present a definite alteration of mitochondrial fusion and oxidative phosphorylation whereas these processes are not significantly affected in haploinsufficient *OPA1‐*mutated fibroblasts.

### Assessment of autophagy and mitophagy in fibroblasts

As activation of the autophagic machinery is essential for the complete degradation of mitochondrial components, we assessed the levels of mitophagy as well as autophagy in *OPA1‐* mutated fibroblasts 34.

We first quantified autophagy levels in *OPA1‐*mutated fibroblasts and controls. The level of autophagy was monitored using two markers, that is the microtubule‐associated proteins 1A/1B light chain 3B (MAP1LC3) (here referred to as LC3) and the sequestosome‐1 or ubiquitin‐binding protein p62 (here referred to as p62). During autophagy, the cytoplasmic form (LC3‐I) is processed into a cleaved and lipidated membrane‐bound form (LC3‐II). LC3‐II is localized in the inner and outer membranes of the phagophore and is essential for membrane biogenesis and closure of the membrane. LC3‐II is re‐cleaved by cysteine protease (Atg4B) following completion of the autophagosome, and recycled 35. P62, a receptor for ubiquitinated proteins, interacts with LC3 to ensure the selective delivery of these proteins into the autophagosome. The accumulation of p62 is an indicator of impaired autophagy 36.

**Figure 3:**
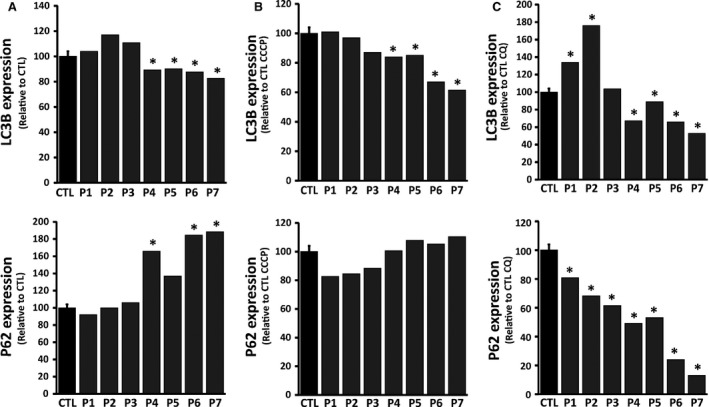
Immunoblot analysis of autophagic response proteins in *OPA1* mutated fibroblasts and controls. Levels of autophagy levels were assessed by quantitative analysis of autophagy markers LC3II and P62 in total cell lysates from cells incubated under normal conditions (DMSO) or pre‐treated with either 60 μM chloroquine (4 hrs) or with 10 μM CCCP (24 hrs) using Western Blot analysis. (**A**–**C**) The results, represented as LC3II conversion (LC3I/total LC3I+II) and P62, show ratios of protein band densities to the densities of the respective tubulin alpha bands (TUBA) (See Fig. S4). To modulate and test efficiency in these cell lines, control and *OPA1* mutated fibroblasts were incubated in presence of either DMSO (**A**), a mitochondrial uncoupler CCCP (**B**), or the autophagy inhibitor chloroquine (**C**). Data obtained from five independent experiments were analysed. P1‐P7: patients; CTL: controls.

The levels of autophagy were assessed under basal conditions and after exposure to uncoupling agents such as carbonyl cyanide m‐chloro phenyl hydrazine (CCCP), which induces mitophagy through its protonophoric activity on mitochondria. The autophagy was greater in dominant‐negative *OPA1‐*mutated fibroblasts (patients 1–3) compared to haploinsufficient *OPA1‐*mutated fibroblasts and controls (patients 4–7) (Fig. 3A). This was reflected by the increased amount of LC3‐II, which has been correlated with the number of autophagosomes 35. Transmission electron microscopy and the quantification of autophagosomes further revealed a greater number of autophagosomes in dominant‐negative *OPA1‐*mutated fibroblasts than in haploinsufficient *OPA1‐*mutated fibroblasts and controls (Fig. S3A and B). In contrast, fibroblasts bearing the I382M mutation in either the mono‐ or the bi‐allelic state showed fewer autophagic vesicles, as reflected by the lower LC3‐II level, than in controls, suggesting a low autophagic response. This was confirmed by the accumulation of P62 in fibroblasts from patients with mono‐ or bi‐allelic *OPA1* mutations, in conformity with various reports on impaired autophagy 36, 37. Indeed, P62 is degraded by the autophagic pathway to prevent the formation of inclusion bodies. Thus, if the pathway functions normally, then there should be no accumulation of P62 36.

We then assessed the consequences of the induction of autophagy using the mitochondrial uncoupler CCCP which collapses the mitochondrial membrane potential to boost the elimination of damaged mitochondria by autophagy. In each of the three genetic backgrounds of *OPA1* mutations, CCCP activated autophagy. The resting autophagy activity being already high in the dominant‐negative *OPA1‐*mutated fibroblasts, the induction of autophagy was less evident in these cell lines (patients 1–3) (Fig. 3B). Upon induction of autophagy, haploinsufficient *OPA1‐*mutated fibroblasts displayed higher LC3‐II levels, demonstrating that the autophagic machinery was functional. However, the conversion of LC3‐II was lower in haploinsufficient *OPA1‐*mutated fibroblasts (patients 4–7), 20% of LC3 remaining in the cytoplasmic form, LC3‐I, compared to dominant‐negative *OPA1*‐mutated fibroblasts and controls in which the induction of autophagy resulted in the full conversion of LC3 into the membrane‐bound form, LC3‐II (Fig. 3B and Fig. S4).

Studies on OPA1(+/−) murine models have shown the greater accumulation of autophagosomes in the RGC layer compared to their wild‐type counterparts 26, 27. The accumulation of autophagosomes results in either an increased activation of autophagy or an impaired degradation of autophagosomes and the recycling of LC3. We therefore analysed the autophagic flux in *OPA1* mutated fibroblasts and controls. Cells were treated with chloroquine (CQ) at 60 μM for 4 hrs (data presented here) and 24 hrs. CQ inhibits the fusion of autophagosomes and lysosomes, thereby blocking the degradation of vesicles and the recycling of LC3‐II 35. This inhibition halts the autophagic machinery and allows assessment of the induction of intrinsic autophagy in cells. We found that treatment with chloroquine for 4 hrs increased the level of the cleaved form of LC3‐II in all fibroblasts with the exception of those from patients harbouring *OPA1* mutations causing the down‐regulation of OPA1. Haploinsufficient *OPA1‐*mutated fibroblasts (patients 4–7) showed no accumulation of LC3‐II compared to dominant‐negative *OPA1‐*mutated fibroblasts (patients 1–3) (Fig. 3C). These results mirror data obtained in resting conditions, which showed low levels of autophagy in haploinsufficient *OPA1* mutated fibroblasts. Recognition of poly‐ubiquitinated proteins by P62 is the first step of the autophagic response. CQ, which serves to evaluate the induction of autophagy, revealed low levels of P62 in haploinsufficient *OPA1‐*mutated fibroblasts. These results combined with data obtained from induction of the autophagic machinery suggest that the autophagy induction is reduced in haploinsufficient *OPA1‐*mutated fibroblasts. Dominant‐negative *OPA1‐*mutated fibroblasts showed an abundant accumulation of LC3‐II upon chloroquine treatment (Fig. 3C). Moreover, P62 levels were similar to those of control fibroblasts, thus reflecting the greater induction of autophagy in dominant‐negative *OPA1‐*mutated fibroblasts. In brief, the analysis of the autophagy flux with CQ, an inhibitor of lysosomal activity, showed that the autophagic machinery is intrinsically activated in dominant‐negative *OPA1‐*mutated fibroblasts as compared to haploinsufficient *OPA1‐*mutated fibroblasts.

**Figure 4:**
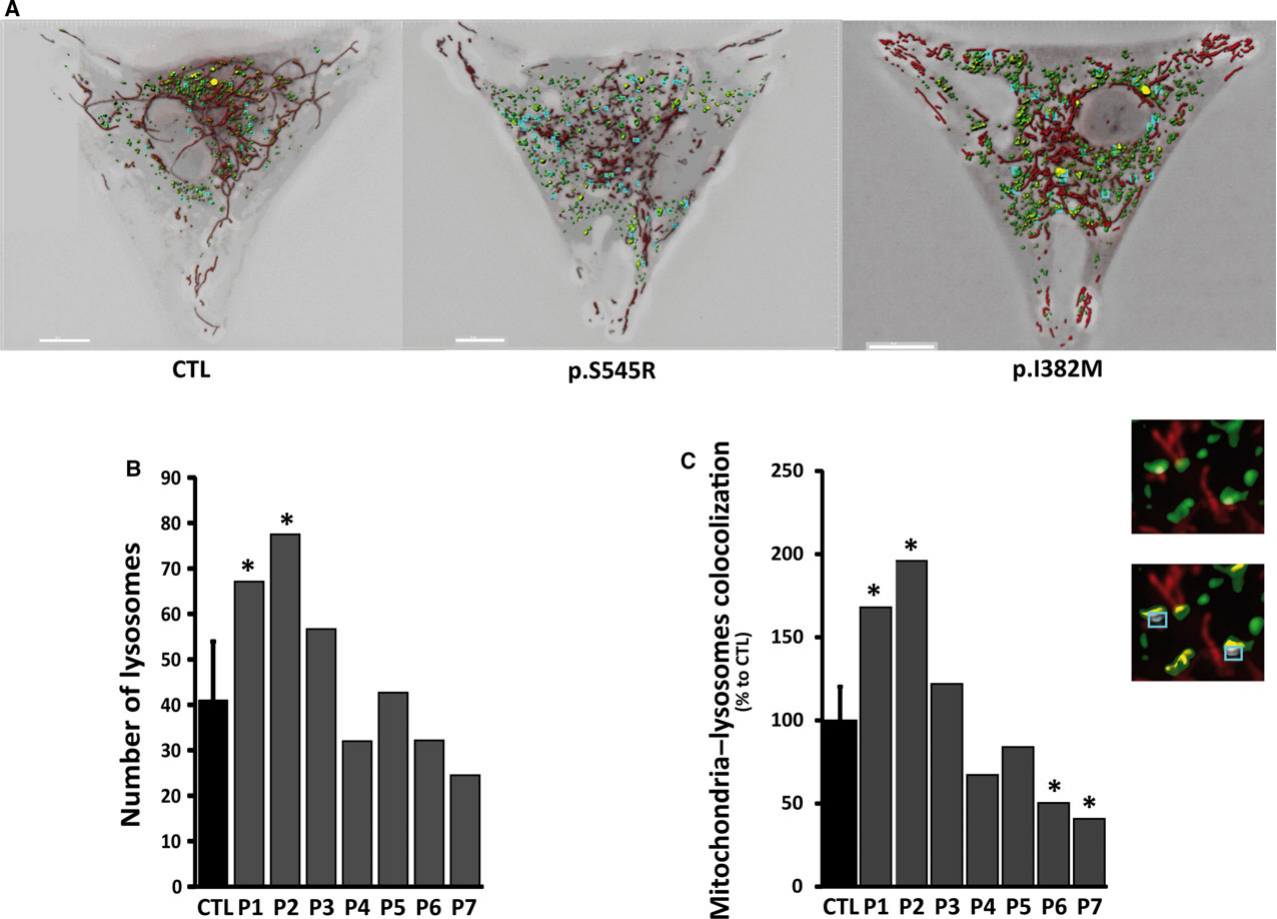
Mitophagy in control and *OPA1* mutated fibroblasts. Before experiments, cells were loaded with Lyso‐ID (green) and MitoTracker Red (red) to visualize lysosomes and mitochondria, respectively. (**A**) The degree of the signal of lysosomes with mitochondria (merge) was calculated *via* cell live imaging microscopy using a Leica Widefield microscope (for details see Materials and methods). Scale bar = 10 μm. **(B**–**C**) The number of lysosomes and mitophagic events were quantified from image acquisitions (For details see Materials and methods). (**C**) Zoomed regions (insets) from the fluorescent merge images (zoomed from the original picture) illustrate the colocalization of lysosomes (green signal) and mitochondria (red signal), appearing as yellow areas indicative of mitophagy *(blue box)*. The graphs are representative of 30 randomly selected cells from at least 3 independent experiments performed in duplicate. P1‐P7: patients; CTL: controls.

Finally, we investigated the consequences of *OPA1* mutations on mitophagy levels in *OPA1* mutated fibroblasts and controls. During mitophagy, the clearance of damaged mitochondria by autophagy, mitochondria are selectively sequestered by autophagic vesicles. We monitored the mitophagic process by direct live cell fluorescence microscopy (Fig. 4A). We assumed that colocalization between the signal for lysosomal structures (Lyso‐ID) and that for mitochondria (MitoTracker Red) represented mitophagic events 35. The signal for lysosomes was higher in dominant‐negative *OPA1‐*mutated fibroblasts (patients 1–3), indicating that the lysosomes were more numerous in these *OPA1* mutants compared to haploinsufficient *OPA1‐*mutated fibroblasts (patients 4–7) and controls (Fig. 4B). As shown in Figure 3, the basal mitophagy reflected by the colocalization of mitochondria and lysosomes was greater in dominant‐negative *OPA1‐*mutated fibroblasts compared to haploinsufficient *OPA1‐*mutated fibroblasts and controls (Fig. 4C). The rate of colocalization between the signal for lysosomes and that for mitochondria was lower in haploinsufficient *OPA1‐*mutated fibroblasts compared to controls, indicating a reduced rate of mitophagy (Fig. 4C). These findings revealed a high mitochondrial turnover rate in dominant‐negative *OPA1‐*mutated fibroblasts in contrast to *OPA1‐*mutated fibroblasts, characterized by the down‐regulation of the OPA1 protein level, in which the autophagic and mitophagic responses were reduced.

## Discussion

The imbalance between mitochondrial fission and fusion induces different autophagic responses. Fusion is rather protective against mitophagy whereas increased fission facilitates mitophagy 29. Studies on *Opa1*(+/−) murine models showed an increase in the number of autophagosomes in the RGC layer of heterozygous mutants compared to wild‐type mice at 24 months 26. *OPA1* defects were hypothesized to impair mitochondrial quality control, thereby rendering cells, particularly RGCs and neurons 15 more susceptible to stress factors 26, 38. In this study, we assessed the mitophagic and autophagic responses, as well as the autophagic flux, in a cohort of fibroblasts of patients carrying various *OPA1* mutations. These included compound heterozygous *OPA1* mutations associating a truncative mutation with the same p.I382M missense mutation in the conserved GTPase domain. This specific pathogenic variant is considered to be highly penetrant for the neurological ‘plus’ features, suggesting that missense GTPase *OPA1* mutations may be potentially more deleterious, possibly *via* a dominant‐negative mechanism and increased mitochondrial DNA instability 39. Our Western blot analysis of fibroblasts harbouring compound heterozygous *OPA1* mutations with the co‐occurrence of the I382M missense mutation and a non‐sense *OPA1* mutation revealed lower levels of OPA1 protein in the mutated fibroblasts than in controls, suggesting that haploinsufficiency contributes to the pathogenesis of the disorder. Our results suggest that missense mutations do not always lead to purely negative dominance. This is further supported by the introduction of the I382M missense mutation in a chimeric construct, which confirmed the haploinsufficient character of this mutation 9. We investigated other missense mutations to assess mitophagic and autophagic efficacy in DOA models. Consistent with previous studies, an assessment of the quantity of OPA1 protein was fairly conserved in fibroblasts from patients carrying the p.S545R or the p.R445H mutations compared to controls. These findings are in agreement with previous reports categorizing these missense mutations as dominant‐negative *OPA1* mutations 14. In brief, our results distinguish two types of *OPA1* mutation: the dominant‐negative *OPA1* mutations, which have no impact on the quantity of OPA1 protein, and the haploinsufficient *OPA1* mutations, due to bi‐ and mono‐allelic I382M mutations, which result in reduced levels of OPA1 protein.

While still under debate, recent studies suggest that the pathogenesis of DOA may be related to the quantity of OPA1 proteins as well as the relative levels of the five isoforms of OPA1 32, 39. Studies on retinal ischaemia–reperfusion (i/r) injuries showed an increase in OPA1 cleavage causing loss of the long OPA1 isoform (long‐OPA1) with the subsequent loss of mitochondrial network connectivity, increased bioenergetics defects, and greater apoptosis. The restoration of long‐OPA1 not only normalized the i/r‐induced changes in mitochondrial morphology but also inhibited the i/r‐induced apoptosis, necrosis, and intracellular ATP loss 40, suggesting that the alteration of the quantitative ratio between the long‐OPA1 and short‐OPA1 may be involved in the pathogenesis of OPA1‐related disorders. The isoform profile of *OPA1* in missense mutations with a dominant‐negative character (p.S545R and p.R445H) was similar to that of controls despite a lower ratio of long‐OPA1 to short‐OPA1. In contrast, fibroblasts harbouring mutations causing haploinsufficiency showed the loss of one short isoform, with a subsequent increase of the ratio of the long isoform/short isoform of OPA1.

Considering the dichotomy of the different *OPA1* mutations, we investigated whether alterations of the profile and quantity of OPA1 protein affected mitochondrial network connectivity and respiration. Functional analysis revealed distinct behavioural patterns of *OPA1* mutations in mitochondrial dynamics and in the OXPHOS function. Assessment of the mitochondrial network morphology in fibroblasts maintained in the glucose media showed that the impact of haploinsufficient *OPA1* mutations on the mitochondrial network organization was milder than that of dominant‐negative *OPA1* mutations, in which the interconnected mitochondrial network gave way to a completely fragmented mitochondrial network. These features have been previously reported in *OPA1* mutants, and the severity of the mitochondrial network alteration in dominant‐negative *OPA1‐*mutated fibroblasts is concordant with the increased expression of short‐OPA1 in these cells. Indeed the overabundance of the short OPA1 isoforms has been linked to abnormal mitochondrial network fragmentation 41.

In our earlier work, we have reported the association of *OPA1* mutations with OXPHOS coupling defects and reduced complex IV activity 19. Our present findings show that the mild mitochondrial uncoupling observed in dominant‐negative *OPA1‐*mutated fibroblasts is compensated by higher electron transport under routine conditions, reflecting the increased use of the respiratory reserve capacity. We found increased OXPHOS protein expression, in particular that of the complex V subunit, in these *OPA1* mutants. We believe that the loss of mitochondrial volume and connectivity, associated with the higher respiratory chain protein expression, leads to an increase in the density of OXPHOS subunits. This in turn may cause the reorganization of cristae in dominant‐negative *OPA1‐*mutated fibroblasts, with subsequent impairment of mitochondrial respiration. Indeed, components of complex V have been shown to play a role in determining the architecture of cristae 42 which in turn directly influences respiratory properties and efficacy 43. The incorporation of ATP synthase dimers in the mitochondrial inner membrane has been found to promote a thermodynamically unstable architecture of cristae 42. In short, dominant‐negative *OPA1‐*mutated fibroblasts show severe fragmentation of the mitochondrial network, loss of mitochondrial mass, and OXPHOS coupling defects.

In contrast, we found that despite a significant decrease of the OPA1 protein level in mono‐ or bi‐allelic p.I382M variants, there was no alteration of mitochondrial respiration. Our findings are further supported by previous studies, which did not find respiratory defects in mitochondria from six DOA patients bearing *OPA1* mutations, among which one patient carried compound heterozygous *OPA1* mutations 44. Moreover, a study on haploinsufficient OPA1(+/−) mouse cardiomyocytes showed that decreased OPA1 expression did not alter the intrinsic mitochondrial function, with no change in mitochondrial oxidative capacity and respiratory chain complex activity 45. In short, *OPA1* mutations causing haploinsufficiency lead to no major alteration of the mitochondrial network, no loss of mitochondrial mass, and no bioenergetic impairment compared to dominant‐negative *OPA1* mutations.

The impairment of OXPHOS and fragmentation of the mitochondrial network are typically associated with the mitophagic removal of damaged mitochondria. We investigated the effect of *OPA1* mutations on mitochondrial quality control. We assessed the mitophagy and autophagy in *OPA1* mutants, as activation of the autophagic machinery is crucial to the complete degradation of mitochondrial components 34. Our assessment of autophagic activity using the LC3 and p62 markers showed that autophagy was greater in dominant‐negative *OPA1‐*mutated fibroblasts compared to haploinsufficient *OPA1‐*mutated fibroblasts and controls. Likewise, the basal mitophagy reflected by the colocalization of mitochondria and lysosomes was greater in dominant‐negative *OPA1* mutants than in haploinsufficient *OPA1* mutants. On the other hand, fibroblast models of *OPA1* haploinsufficiency, which do not involve drastic alterations of the mitochondrial network, showed lower mitophagic activity and fewer autophagic vesicles, as reflected by lower LC3‐II levels, indicating a low autophagic response. This is confirmed by the accumulation of P62, which has been shown to reflect impaired autophagy 37. Autophagic flux analysis using chloroquine, an inhibitor of lysosomal activity, showed that the autophagic machinery is intrinsically activated in dominant‐negative *OPA1* mutants but not in haploinsufficiency *OPA1* mutations. Recent studies have shown that the stimulation of OXPHOS enhances mitochondrial renewal by increasing the rate of mitochondrial degradation 46. We believe that mitochondrial uncoupling promotes the high mitochondrial turnover rate in dominant‐negative *OPA1* mutations. In dominant‐negative *OPA1‐*mutated fibroblasts, OXPHOS uncoupling mimics high mitochondrial activity thereby initiating mitophagy. In contrast, in haploinsufficient *OPA1‐*mutated fibroblasts, the low response is mainly due to the absence of stimuli. When mitochondrial damage is experimentally induced by uncoupling agents, such as CCCP, autophagy and mitophagy are exacerbated in haploinsufficient *OPA1‐*mutated fibroblasts, thus demonstrating the functional operation of the autophagic machinery.

We hypothesize that the reduction of autophagy and mitophagy, particularly the absence of stimulation of these pathways, may be part of the pathophysiology of *OPA1* mutations leading to haploinsufficiency. This is in agreement with recent reports documenting the alteration of mitophagic and autophagic activities in *OPA1*‐related diseases 12, 47. Studies on *in vitro* neuronal models and an *in vivo* mouse model of OPA1 insufficiency have shown that the down‐regulation of OPA1 reduces the level of BNIP3 (BCL2/adenovirus E1B 19 kDa interacting protein 3) 47, previously implicated in the induction of mitophagy and autophagy 48. While the reduction of mitophagy and autophagy is not itself sufficient to induce spontaneous apoptotic cell death, it contributes greatly to the susceptibility of neurons to chronic and acute stress 28. These reports, taken together with our findings, suggest that the OPA1 protein level may impinge on the levels of mitophagy and autophagy. Our hypothesis is further reinforced by studies showing that mitophagy may constitute a pro‐survival pathway through the up‐regulation of OPA1 expression 49. However, a recent communication on bi‐allelic *OPA1* mutations reports an increase of basal mitophagy, associated with excessive fragmentation of the mitochondrial network, in biallelic and monoallelic patients with DOA ‘plus’, as well as in OPA1 siRNA‐treated control cultures 50. The results reported by the authors on haploinsufficient *OPA1* mutations correspond closely with our own findings on dominant‐negative *OPA1* mutations. In contrast, our study showed no major alteration of the mitochondrial network in fibroblasts harbouring bi‐allelic *OPA1* mutations. This discordance may be due to the fact that we used a pooled sample of bi‐allelic cases carrying the p.I382M mutation, and whereas the p.I382M mutation seems to impact OPA1 quantitatively, it affects only moderately the pro‐fusion activity of OPA1. Indeed, perturbation of the dynamic cycle of mitochondrial fission and fusion is likely to dysregulate mitophagy, which is driven by an excess of fragmented mitochondria. In addition to their involvement in the mechanism underlying DOA, whether negative dominant or haploinsufficient, a common feature of *OPA1* mutations is their potential impact on the mitochondrial network and the subsequent increase of the mitophagic response. Taken together, these findings suggest that besides the type of *OPA1* mutation and quantity of OPA1, the alteration of the pro‐fusion activity of OPA1 may drive the mitophagic response observed in some severe cases of DOA.

**Figure 5:**
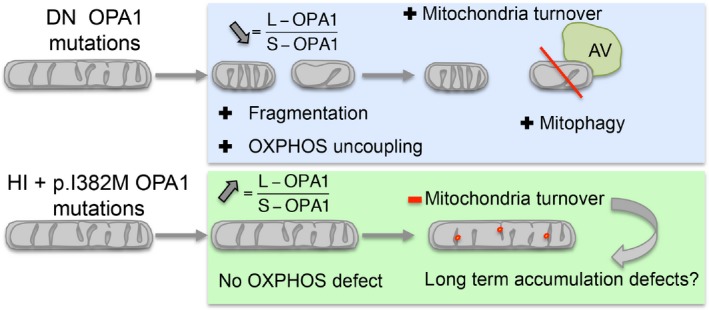
Mitophagy regulation in DOA+. DOA, dominant optic atrophy; DN, Dominant Negative; HI, HaploInsufficient; AV, Autophagic vesicle.

In conclusion, our functional analysis showed distinct alterations of mitochondrial physiology and turnover depending on the type of *OPA1* mutation involved (Fig. 5). We believe that in addition to the mitophagic and autophagic responses acting as modulating factors in the pathogenesis of DOA, the OPA1 protein level and alteration of its fusion activity may also impinge on the severity of the disorder. The interference of the *OPA1* mutation in protein function, which appears to determine mitochondrial quality control, may thus be implicated in the neurodegeneration that extends beyond the retinal ganglion cell. Our work highlights the necessity of developing specific therapeutic strategies in diseases related to defective mitochondrial dynamics.

## Conflict of interest

The authors declare no conflict of interest.

## Supporting information


**Figure S1** Mitochondrial network fragmentation in control and *OPA1* mutated fibroblasts.

**Figure S2** Mitochondrial mass in *OPA1* mutant fibroblasts and controls.

**Figure S3** Transmission electron microscopy pictures of autophagic vesicles (white arrows).

**Figure S4** Representative western blots of Figure 3.

**Movie S1** A representative cell with p.I382M and p.R824* mutations analysed by IMARIS.
