# Supplementary figures and images for: Autophagy controls the pathogenicity of OPA1 mutations in dominant optic atrophy
Source: J Cell Mol Med. 2017 Apr 4;21(10):2284–97. doi: 10.1111/jcmm.13149 (PMC5618673; doi:10.1111/jcmm.13149)

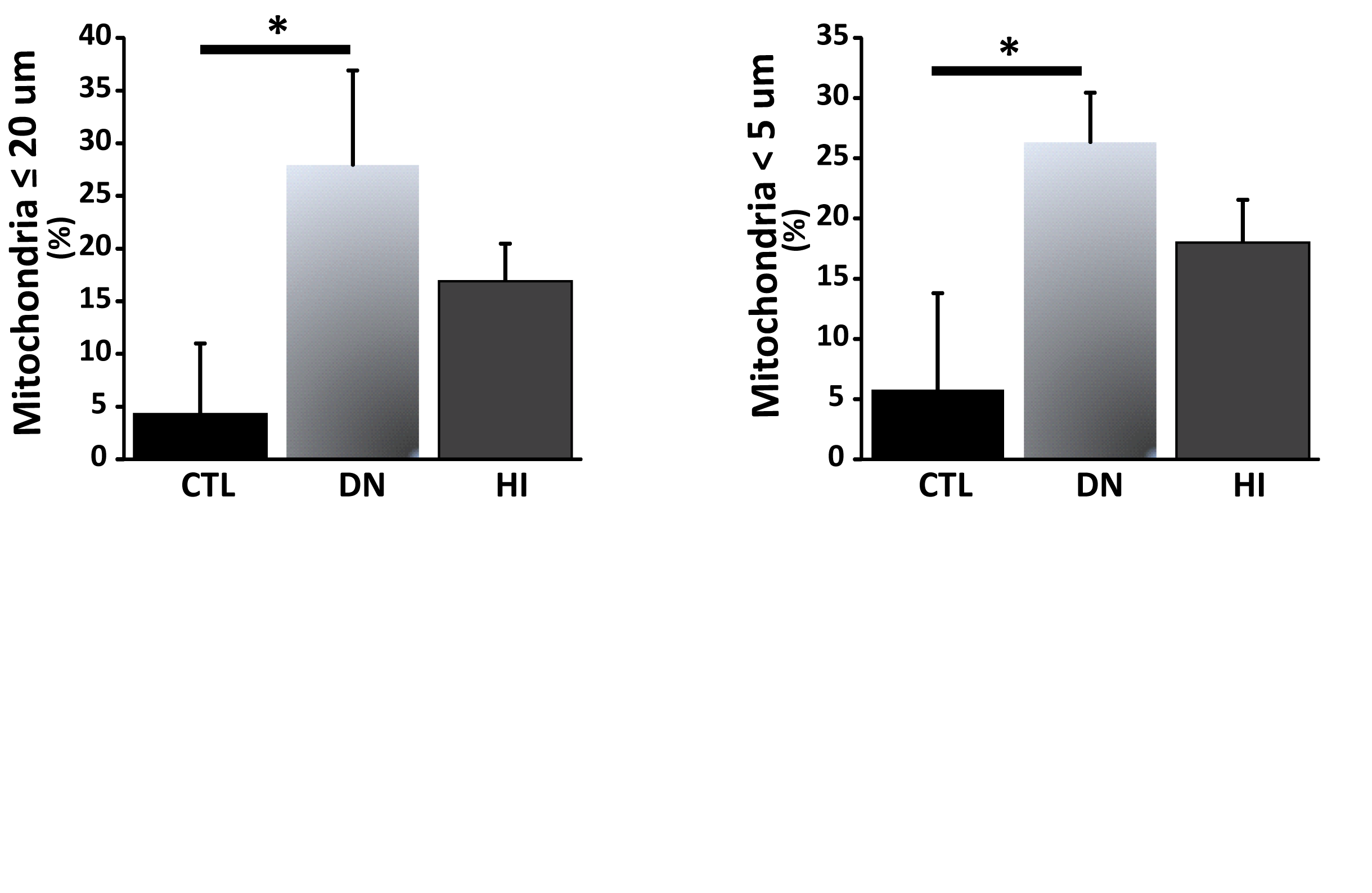

Supplement: Supplementary file 1 — Figure S1 Mitochondrial network fragmentation in control and OPA1 mutated fibroblasts. [file JCMM-21-2284-s001.tif]

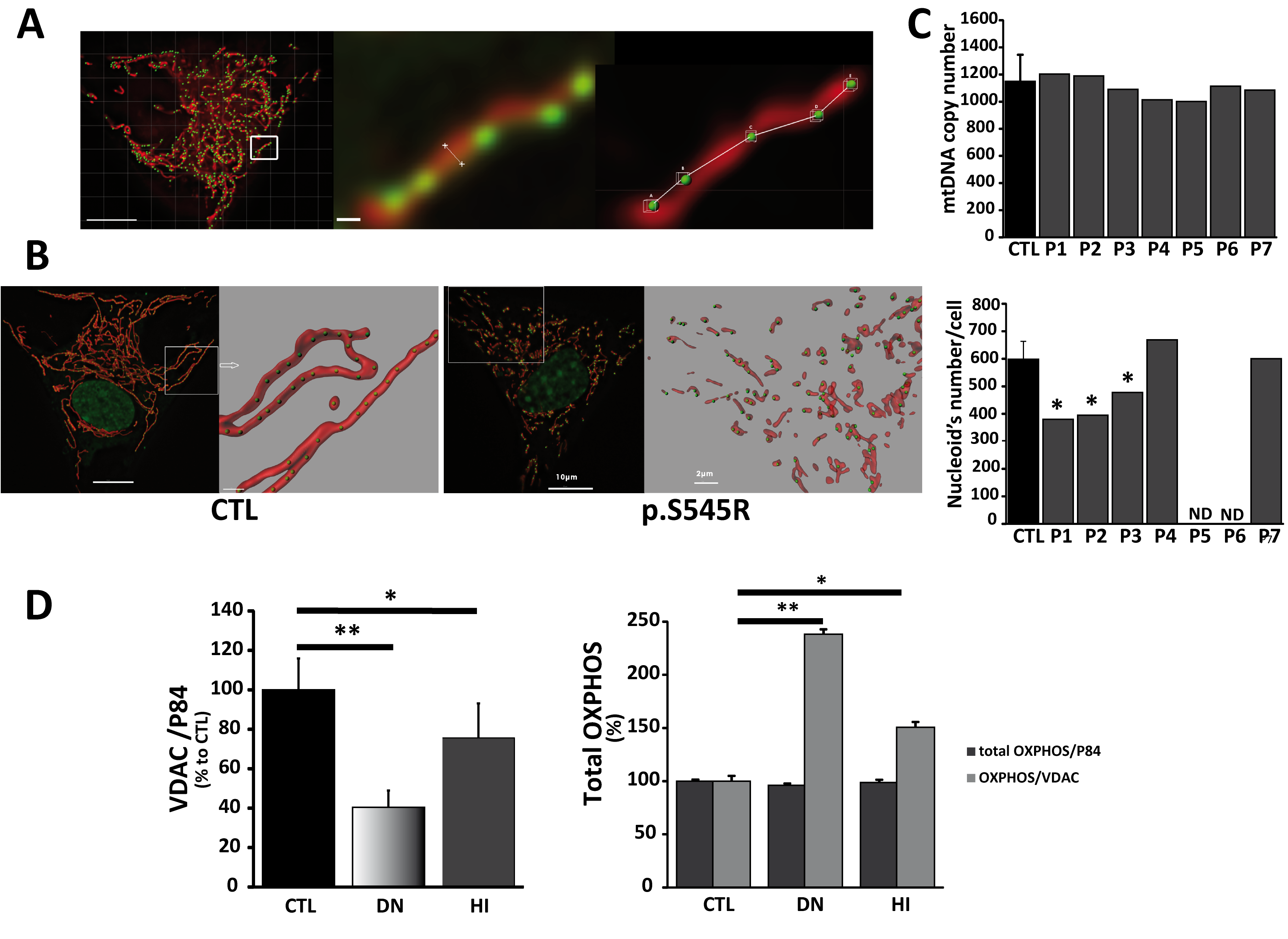

Supplement: Supplementary file 2 — Figure S2 Mitochondrial mass in OPA1 mutant fibroblasts and controls. [file JCMM-21-2284-s002.tif]

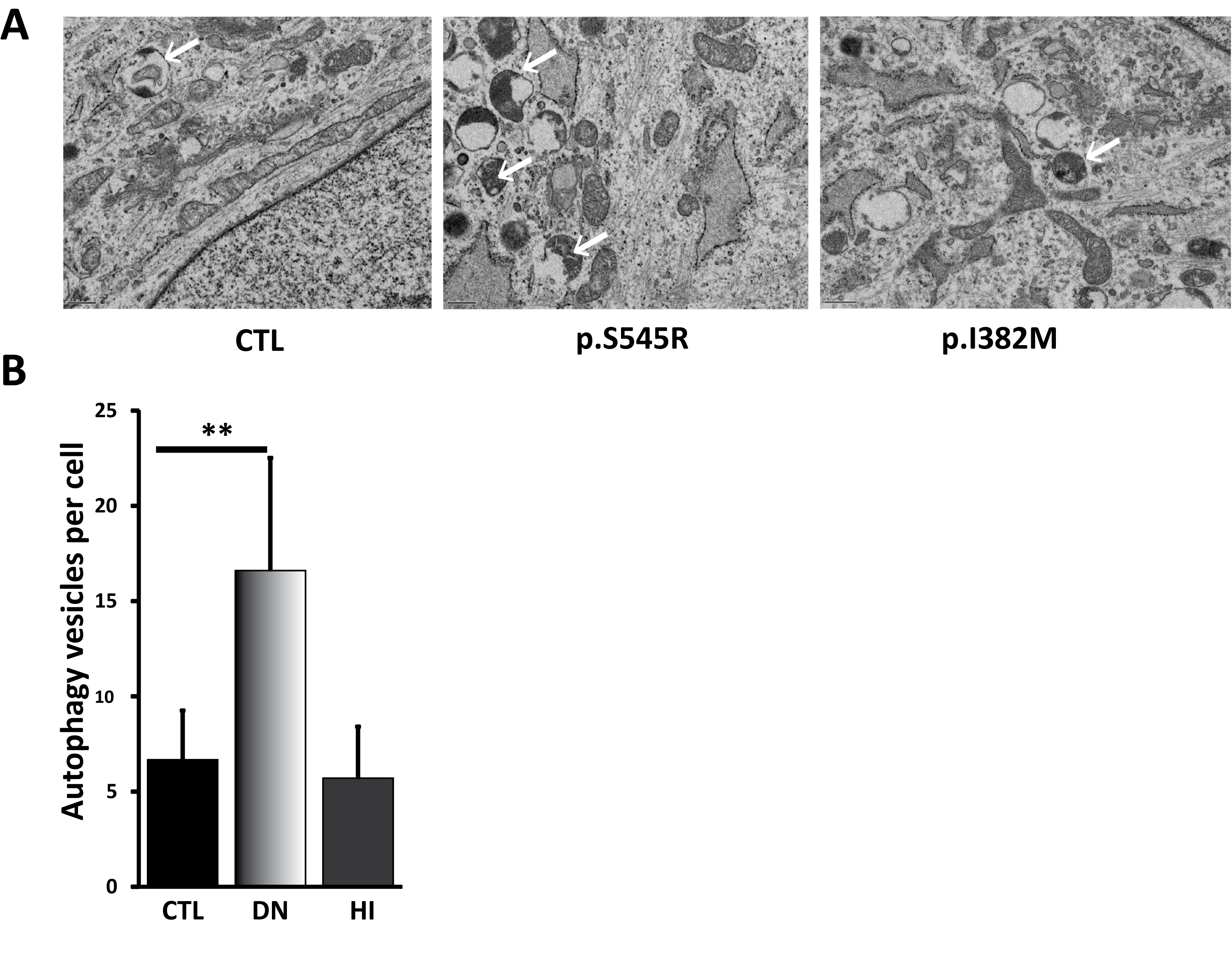

Supplement: Supplementary file 3 — Figure S3 Transmission electron microscopy pictures of autophagic vesicles (white arrows). [file JCMM-21-2284-s003.tif]

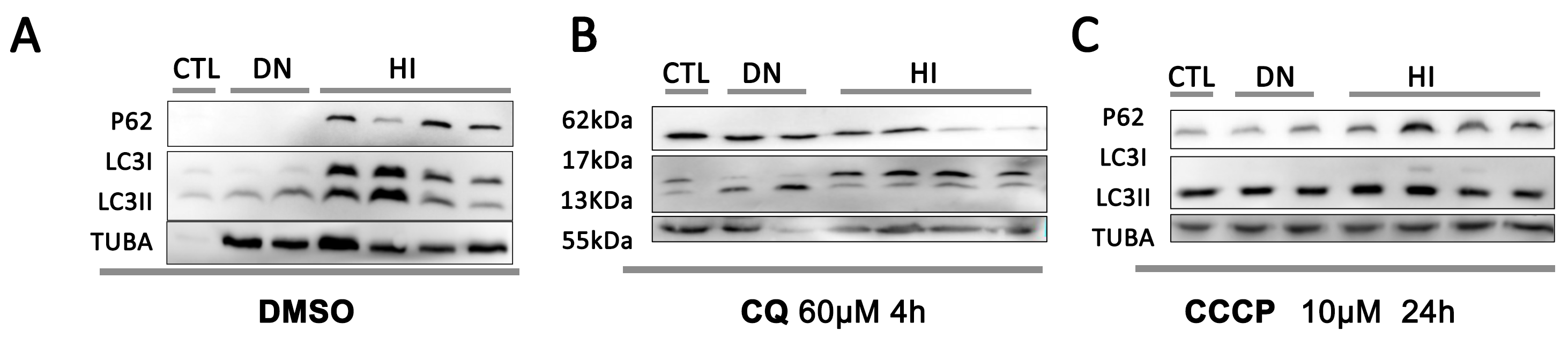

Supplement: Supplementary file 4 — Figure S4 Representative western blots of Figure 3. [file JCMM-21-2284-s004.tif]
